# Dual Role of miR-122 in Molecular Pathogenesis of Viral Hepatitis

**DOI:** 10.5812/hepatmon.6128

**Published:** 2012-05-30

**Authors:** Hossein Sendi

**Affiliations:** 1 The Liver-Biliary-Pancreatic Center, Cannon Research Center, Carolinas Medical Center, Charlotte, USA; 2 Department of Biology, University of North Carolina at Charlotte, Charlotte, USA

**Keywords:** Micro RNAs, Hepatocyte Nuclear Factors, Cyclin G1

## Abstract

The hepatic microRNA (miRNA), miR-122, is the most abundant miRNA within the liver, where it accounts for 70% of the total miRNA pool. It is known that miR-122, as an unusual host factor, increases the abundance of hepatitis C virus (HCV) RNA in HCV infection by binding directly to the 5’-UTR of the viral genome. Therefore, it has been suggested as a potential target for the treatment of hepatitis C. However, recent evidence shows that miR-122 decreases HBV replication through the inhibitory effect of p53 on HBV transcription, and consequently it acts as a tumor-suppressor through both a decrease in HBV replication and by directly targeting cyclin G1, as well as Wnt/beta-catenin, and NDRG3 pathways. This paper will brieﬂy discuss the underlying mechanisms for the dual role of miR-122 in viral hepatitis, and explains why therapeutic applications of miR-122 may differ based on the underlying disease.

MicroRNAs (miRNAs) are approximately 22 nucleotide non-coding RNAs that can down-regulate products of different genes through either cleavage or a reduction in their translational eﬃciency of the target mRNAs [1]. In the nucleus, pri-miRNA which are double stranded RNA segments, approximately 60-70 nucleotides in length, are cleaved by the RNAse III enzyme, Drosha. This makes hair-pin shaped double stranded RNAs (pre-miRNAs) which are transported by exportins to the cytoplasm. In the cytoplasm, Dicer which is also an RNAse III enzyme,cuts them further into mature miRNAs. Finally, binding of the miRNA to the RNA-induced silencing complex (RISC) leads to translational repression or degradation of the target mRNA [1]. miR-122, is a 22 nucleotide miRNA that comprises 70% of the total miRNA population in normal adult hepatocytes with approximately 66 000 copies per cell [2]. The most well-known function of miR-122 in the mammalian liver is to regulate lipid and cholesterol metabolism. The knockdown of miR-122 expression, down-regulates cholesterol and lipid metabolizing enzymes and reduces plasma cholesterol levels [3].The importance of miR-122 in HCV infection was ﬁrst identiﬁed when Jopling et al. found that miR-122 was expressed in human hepatoma cells (Huh7), which are permissive to HCV replication, but not in a different human liver cell line, HepG2 which does not support HCV replication [4]. HCV RNA was reduced by about 80% when miR-122 was silenced in the Huh7 cells, stably expressing genotype 1b HCV replicon [4]. The effect of miR-122 on HCV is based on its binding to the 5’ UTR of HCV RNA [4]. This identiﬁed miR-122–binding site is in an unstructured region of the 5’ UTR, upstream of the HCV internal ribosome entry site (IRES), and it is conserved across all six HCV genotypes [5]. This ﬁnding showed that miR-122 is important for the eﬃcient replication of HCV RNA in vitro. Because these authors did not observe any effect of miR-122 binding on HCV translation or RNA stability, they concluded that miR-122 positively regulates HCV at the level of viral replication [4]. A second binding site for miR-122 was identiﬁed adjacent to, and downstream of, the ﬁrst one. It was shown that miR-122 binds to both sites within the same HCV molecule increasing the abundance of HCV RNA [5]. However, later it was shown that miR-122 does not directly stimulate HCV RNA synthesis [6][7]. The fact that binding sites of miR-122 were located directly upstream of an internal ribosome entry site (IRES) brought up the second theory that miR-122 can stimulate viral protein translation [8][9]. However, translation enhancement only partially explains the role of miR-122 in the HCV life cycle [9][10]. Interestingly, in another recent study, Shimakami et al. showed that miR-122 binds HCV RNA in association with Argonaute2 (Ago2), and that this slows decay of the HCV RNA in infected cells [10]. These observations roused much interest in the role of miR-122 in HCV infection and its potential as a therapeutic target. Accordingly, Lanford et al. showed that silencing of miR122 in chronically HCV-infected chimpanzees led to long-lasting suppression of HCV viremia [11]. However, when the miR-122 level was sought in patients with chronic hepatitis C (CHC), different results were obtained. In one study miR-122 levels were examined in patients with different responses to Interferon (IFN) treatment, and no positive correlation was detected between intrahepatic miR-122 and HCV RNA levels [12]. In this study, those subjects who were non-responder to IFN were found to have pretreatment levels of miR-122 several times lower than those who responded [12]. The lack of correlation between miR-122 and HCV RNA levels was conﬁrmed in another recent study, in while miR-122 levels was found to be strongly associated with serum ALT and with necroinﬂammatory activity in patients with CHC [13].

In contrast to hepatitis C, there is new evidence that highlights an anti-viral role for miR-122 in hepatitis B. In a recent study, cyclin G1 was found to be a direct target of miR-122 [14]. Thus, the authors suggested that miR-122 down-regulation, consequent to HBV infection, leads to up-regulation of cyclin G1, which initiates formation of a cyclin G1-p53 complex [14]. They speculated that the subsequent release of p53 from binding to HBV enhancers, facilitates HBV mRNA transcription [14]. The result of this study would be more plausible if combined with the results of another recent study which shows that the over-expression of cyclin G1 increases Akt activation [15]. This, in turn leads to subsequent phosphorylation of GSK-3β and stabilization of Snail, a critical epithelial-mesenchymal transition (EMT) mediator. They also found a signiﬁcant correlation between the expression of cyclin G1 and p-Akt levels in a cohort of patients with hepatocellular carcinoma (HCC) [15]. Therefore, they suggested that cyclin-G1 may serve as a novel prognostic biomarker and therapeutic target [15]. Taken together, down-regulation of miR-122 consequent to HBV infection can lead to the over-expression of cyclin-G1. This, in turn not only leads to an increase in HBV replication, but it also leads to an increase in Akt activation and the subsequent initiation of epithelial-mesenchymal transition. These additive/ synergistic outcomes of miR-122 suppression in the context of chronic HBV infection may predispose patients to an increased risk of HCC development and progression. Therefore, miR-122 as well as cyclin-G1 [15] may serve as a novel prognostic biomarker and have some therapeutic applications in HBV-induced HCC.

As mentioned above, it is known that miR-122, as an unusual host factor, enhances HCV replication by binding to two closely spaced target sites in the 5’-UTR of the viral genome, which leads to an increased abundance of HCV RNA [4][5]. In contrast, new ﬁndings highlight an anti-viral as well as tumor-suppressive role for miR-122. The anti-proliferative properties of miR-122 have been reported in other recent studies as well [16][17][18]. Although, miR-122 was found to be suppressed in chronic HBV infected patients, the mechanism is still unclear. The antiviral role of miR-122 is suggested by the inhibitory effect of p53 on HBV transcription through blocking of the binding of transcription factors like hepatocyte nuclear factors (HNFs) to HBV enhancers [14]. Worthy of note, is that it has been shown that hepatocyte nuclear factors like HNF3 and HNF4α regulate miR-122 expression in hepatocytes [19][20]. Therefor both HBV and miR-122 need HNFs for enhancement of their genome transcription. It is possible that in chronic HBV infection, HBV utilizes HNFs for its own transcription rather than using these factors for transcription enhancement of miR-122, and this in turn leads to the suppression of miR-122. It is also interesting to mention that we have identiﬁed a novel double HBV core promoter mutation, which is speciﬁc and common in genotype D of HBV isolates in patients with chronic hepatitis B and this creates a new binding site for HNF3 [21]. This may suggest that mutation variability in different genotypes of HBV might be explained by different levels of expression in miR-122 in selected contexts of host genetic factors and HBV genotypes. The role of hepatocyte nuclear factors in the co-regulation of HBV and miR-122 still needs further clariﬁcation.

In summary, miR-122 plays a dual role in the molecular pathogenesis of viral hepatitis. While it increases the abundance of HCV RNA in HCV infection by binding directly to the 5’-UTR of the viral genome, it decreases HBV replication through an inhibitory effect of p53 on HBV transcription, and consequently it acts as a tumor-suppressor through both decreasing of HBV replication and directly targeting cyclin-G1, as well as Wnt/β-catenin, and NDRG3 pathways. Thus, while the silencing of miR-122 has been suggested as a novel therapeutic approach in combating HCV infections, on the ﬂip side miR-122 mimics may be considered as a novel therapeutic candidate for HBV infection and HBV-induced HCC. Taken together, while the advantages of using miR-122 inhibitors include decreasing in plasma cholesterol or HCV RNA levels in the context of hyper-lipidemia or hepatitis C, the beneﬁts of using miR-122 mimics include tumor suppressive effects and decreasing in HBV replication in the context of hepatitis B. Using either miR-122 inhibitors or mimics needs to have further careful evaluation considering the potential risks of oncogenesis or hyper-lipidemia, respectively. The results of these interesting studies coupled with further studies will doubtless help us to unravel the still largely unknown mechanisms of the actions of miR-122, the most abundant and still mysterious hepatic micro-RNA.
